# Biomechanics of accurate and inaccurate goal-kicking in Australian football: Group-based analysis

**DOI:** 10.1371/journal.pone.0241969

**Published:** 2020-11-11

**Authors:** Stephanie Blair, Sam Robertson, Grant Duthie, Kevin Ball

**Affiliations:** 1 Institute for Health and Sport (IHES), Victoria University, Melbourne, Australia; 2 English Institute of Sport, Manchester, England; 3 School of Exercise Science, Australian Catholic University, Sydney, Australia; Federation University Australia, AUSTRALIA

## Abstract

Goal-kicking is an important skill in Australian Football (AF). This study examined whether kinematic differences exist between accurate and inaccurate goal-kicks and determined the relationships between technical factors and accuracy. Eighteen elite to sub-elite AF players performed 15 x 30 m goal-kicks on an AF training ground, with three-dimensional kinematics collected using the Xsens inertial measurement system (Xsens Technologies B.V., Enschede, the Netherlands). A general linear mixed modelling approach and regression-based statistics were employed to quantify differences between accurate and inaccurate goal kicks and the relationships between technical factors and accuracy. Accurate goal-kicks were characterised by a straighter approach line, with less kick-leg joint range of motion (knee and hip), lower linear velocity (centre of mass, foot speed), angular velocity (knee and shank), and less support-leg knee flexion during the kicking phase compared to inaccurate goal-kicks. At the end of the follow through, players produced greater ankle plantarflexion and a straighter-leg line in accurate goal-kicks. Findings in this research indicated that many factors interact with goal-kicking accuracy in AF, ranging from the players’ approach line path, their support-leg mechanics, the kick-leg swing motion, to the final position of the kicker during their follow through.

## Introduction

Goal-kicking forms an important component of winning games in Australian Football (AF), as it provides a means through which to score points [1]. There are two broad categories of goal-kicking in AF: general play and set-shot goal-kicks. The set-shot is of particular importance, as it comprises approximately 62% of points scored during a game and has been identified as the most influential performance indicator in match outcome [1, 2]. As the success rate for goal-kicks in the 2019 Australian Football league season was only 45.8% (Champion Data statistics, 2019), there is clear scope for research to explore set-shot goal-kicking to support improvements in performance.

The set-shot (hereafter, referred to as the ‘goal-kick’) is a self-paced closed skill, where the player has 30s to perform the shot without any physical pressure from opponents [3, 4]. It is frequently performed using a drop-punt kick, and involves the combined technical aspects of a running approach, release of the ball from the hands, and a forceful impact with the foot of the kick-leg as it swings through in the direction of the goals [5–7]. As the ball is in projectile motion after it leaves the foot, one of the main possible reasons for the kick to miss the goal, is due to a technical error that leads to a poor impact with the ball [3, 7, 8].

Despite the importance of goal-kicking in AF, only two studies have examined the biomechanics of the skill [5, 9]. In an in-field notational analysis of goal-kicking in eight elite AF players, Ball et al. [5] found accurate kickers adopted a straighter approach line, dropped the ball in line with the kicking thigh and finished with the kick-leg pointing towards goals. Whilst notational analysis provided an initial understanding of the influence of specific parameters on goal-kicking performance, it was limited to a front plane analysis of the movement. As the goal-kicking action is a linear movement, important technical characteristics may also occur in the sagittal plane, requiring further investigation. In an in-field examination of 20m goal-kicks in two junior AF players, accurate kicks were associated with greater support-leg (>4°) and kick-leg (>3°), knee flexion [9]. Blair et al. [9] suggested further work was required in a larger sample to establish statistically significant results, making the information more generalisable to the AF population.

Research examining accuracy in punt-kicking in other tasks is also limited. When elite AF players (n = 12) kicked towards a 15 m target (task representative of kicking to a player), accurate kickers produced greater support knee flexion (> 5.3°), hip flexion (> 5°), with greater anterior pelvic tilt (+8.1) [10]. The authors suggested this might be a strategy to improve stability through lowering COM, to improve accuracy. Peacock et al. [11] found that when elite AF players (n = 11) kicking for accuracy (20m kick to a player), players exhibited lower ankle plantarflexion and higher ankle ROM compared to when kicking for maximal distance. This was suggested to be a mechanism to help achieve a flatter ball flight trajectory, and in turn, improve the accuracy of the kick.

Expanding upon these studies and investigating the complete goal-kicking action is needed to provide a more comprehensive understanding of the technical elements that may be important for improving goal-kicking performance. This knowledge is important as it can be used to objectively guide development programmes aimed at improving goal-kicking, as well as providing readily usable coaching cues [7]. Therefore, the aims of this research was to compare and identify if kinematic differences exist between accurate and inaccurate goal-kicks, and examine the relationships between technical factors and goal-kicking accuracy.

## Methodology

### Participants

Eighteen male AF players (age: 17.4 ± 0.5 yrs; height: 184.5 ± 5.4 cm; mass: 73.1 ± 6.9 kg) volunteered to participate in this research. Players ranged in skill level from elite (AFL Academy squad, the highest squad level available to 16–18 year old players) to a sub-elite cohort and in playing position (full forwards, half forwards and centre line players). Players were selected based on game demands (coaches identified players that regularly performed the goal-kick during a match) rather than playing level, to represent a higher skilled cohort of goal-kickers [12]. All players where competing regularly in competition and had no lower extremity injuries in the previous six months. Ethical approval (HRE17-046) was granted from the Victoria University Human Research Ethics Committee and written informed consent was obtained from each player, and where appropriate a parent or guardian.

### Equipment

Kinematics (240 Hz) were collected using the Xsens MVN link inertial measurement system (IMS) (Xsens Technologies B.V., Enschede, the Netherlands), which has been previously validated to measure kicking kinematics in AF [13]. The system is composed 17 inertial sensors, a transmission pack and battery, zipped into a compression suit which is worn by each player. Each sensor integrates a 3D accelerometer (scale: ± 160 m.s^-2^, noise: 0.003 m.s^-2^/√Hz), 3D gyroscope (± 2000 °/s, 0.05 °/s/√Hz) and 3D magnetometer (± 1.9 Gauss, 0.15m Gauss/√Hz). Sensors were placed on both feet (lateral side of the boot), shanks (medial surface of the tibias), thighs (lateral side above the knees), pelvis (middle of both the posterior superior iliac spines), shoulders (middle of the scapula spine), upper arms (lateral side above elbow), forearms (lateral and flat side of wrist), hands (posterior side), sternum and back of the head [13, 14]. The tightness of the Xsens suit was maximised for each individual to reduce underlining soft tissue artefact and sensor movement [14]. Anthropometric measures were collected from each participant to scale the Xsens biomechanical model (cm); body height, shoulder height, arm span, shoulder width, leg length, knee height and hip width. System calibration was made via a static (N-Pose) and a dynamic (walking) procedure (MVN Analyze 2018). As the IMS is unable to identify the location of the goal-posts during data collection, the N-pose calibration was performed directly in-front of the goals in the centre. This enabled identification and calculation of the goal-centre during data analysis through creating a virtual laboratory segment 30 m away from the global laboratory axis.

### Testing protocol

The testing venue was the regular training and playing ground for the players. Testing was conducted using new footballs (size 5, Sherrin, Australia; official ball in AF competition), inflated within the specified pressure range of 67–75 kPa [6]. Testing was performed during low wind and dry conditions.

**Fig 1:**
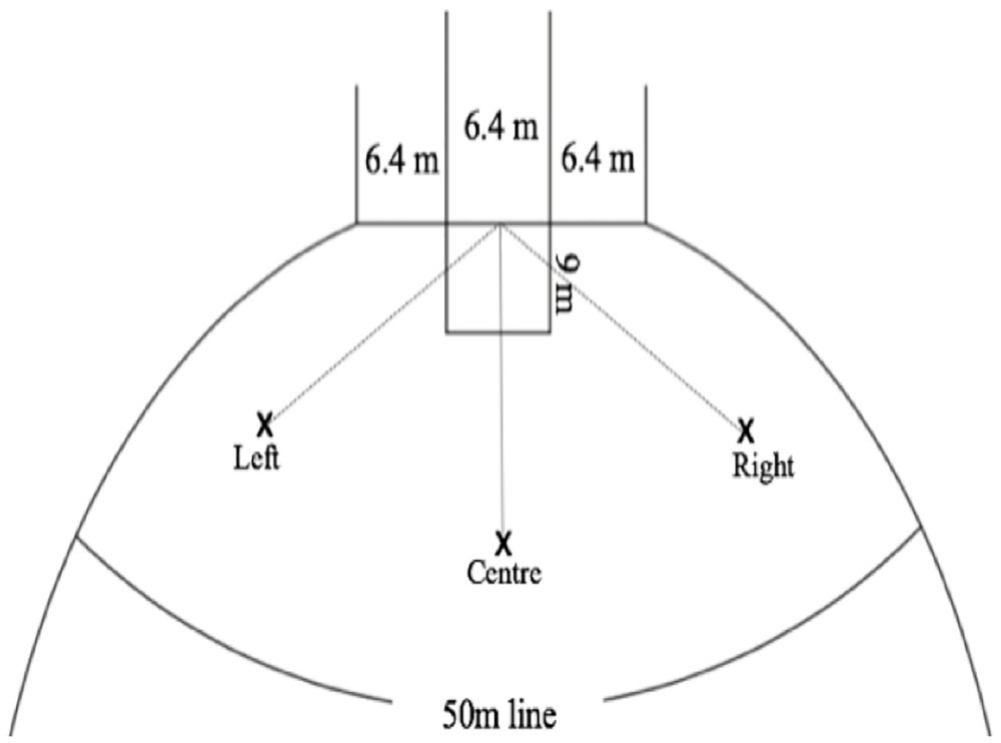
Schematic of the experimental set-up; each kicking position was 30 m from the goal and kicks taken at the right and left positions were at a 45-degree angle to the goal. Goal-kicking positions were representative of typical positions used in competition (as identified by Champion Data from the 2017 AFL season).

All players performed a standardised warm-up, comprised of phases of running-based activities, interspersed with static and dynamic stretching, followed by a minimum of 10 familiarisation goal-kicks from different positions in-front of goals. Players were then instructed to perform 15 x 30 m goal-kicks from three different positions in-front of goals (Fig 1). Two cones were placed on the ground to define the location from where players were required to perform the goal-kick. Players were asked to perform goal-kicks under game-like conditions, including the 30s period players are given to perform this kick from when the mark is taken. All players used a self-selected run-up and performed kicks using their preferred kicking foot. The order of kicking positions was randomised to prevent order and sequence effects, and players were given a 1-minute rest period between trials to avoid the possible influence of fatigue [15].

**Fig 2:**
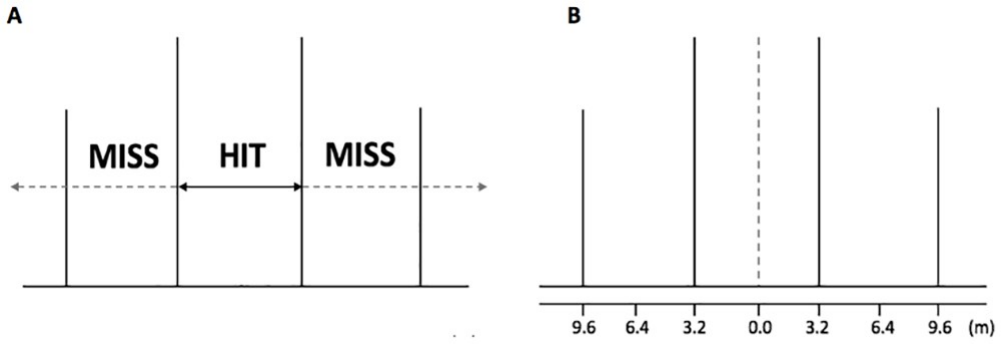
Accuracy grading; a) hit vs miss and, b) lateral horizontal distance measured from the centre of goals (m).

Accuracy was measured using two criteria: 1) hit vs miss [9, 10, 16, 17], and 2) by measuring the horizontal distance from the goal centre [12, 18] (Fig 2). Each kick outcome was recorded manually by the same investigator as the ball crossed the goal-line, using a measuring tape. The hit (accurate) vs miss (inaccurate) method represents a true performance measure which corresponds to how goal-kicks are classified in competition and provides a discrete measure of performance to enable statistical comparisons to made between accurate and inaccurate goal-kicks [9, 19]. The use of the horizontal distance method provides a continuous measure of performance, enabling the examination of the strength of the association between technical parameters and accuracy through regression-based statistics [19].

### Data analysis

Sensor fusion was made using the Xsens Kalman Filter in MVN Analyze 2018. The Xsens biomechanical model was assigned to motion files in Visual 3D and model-based calculations were computed using the Y-X-Z Cardan sequence (ML- AP—Axial rotations). To avoid measurement issues that exist when analysing kinematic data across impacts, no evaluation of the impact phase was performed [20] and parameters during the kicking phase were analysed until the instant before ball contact (BC) and follow-through parameters were analysed separately [21]. Toe-off (TO) corresponded a peak in the gyroscope signal from the foot sensor and BC corresponded to the instant prior to a peak in the anterior-posterior and vertical acceleration signal from the kick-foot sensor [13, 21]. A description of all parameters is provided in Table 1. For kicks taken at an angle, a virtual axis was created to correct the principle axes and align it with the direction of goals (aligned with the direction of progression), computed via the position of the origin of the pelvis at each position utilising method 2 recommended in the visual 3D WIKI documentation. Sagittal plane ankle, knee and hip joint angles were calculated as anatomical angles, with the knee measured as the angle between the thigh and shank and the pelvis used as the coordinate systems for the hip. Pelvis, thigh, shank and foot segment angles were calculated in relation to the global axis. Range of motion (ROM) parameters were calculated as the differences between the angle maxima and minima from the top of backswing to the instance before BC.

**Table 1:** Definitions of technical parameters calculated in this study.

Parameter	Definitions
*Approach phase*	
Approach angle (°)	Angle between start of approach and start of the kicking phase (0° indicates a straight line with an increasing angle indicating a curved approach)
COM velocity (m.s^-1^)	Velocity of the centre of mass
Last step distance (m)	Distance between the heel of the kick foot when in contact with the ground to the toe of the support foot when in contact with the ground.
*Kicking phase*	
**Linear velocities (m.s**^**-1**^**)**	**Linear velocity measured at the instance prior to BC of kick-leg joints/segments**
Foot speed	Velocity of the centre of mass of the foot segment
COM velocity	Velocity of the centre of mass
**Angular velocities (°/s)**	**Angular velocities of the kick leg measured at BC and maxima**
Ankle angular velocity	Angular velocity of the ankle (represents plantarflexion)
Knee angular velocity	Angular velocity of the knee (represents extension)
Shank angular velocity	Angular velocity of the shank segment about the global y-axis
Thigh angular velocity	Angular velocity of the thigh segment about the global y-axis
Hip angular velocity	Angular velocity of the hip (represents flexion)
**Range of motion (°)**	**Differences between angle maxima and minima during forward swing phase**
Ankle ROM	Ankle joint (flexion/extension)
Knee ROM	Knee joint (flexion/extension)
Hip ROM	Hip joint (flexion/extension)
Pelvis ROM	Pelvis angle about the global y-axis
**Joint angles (°)**	**Joint angles for the kick-leg and support-leg, (at BC, Maxima and SHS)**
Ankle angle	Angle between the foot and shank, plantar-dorsi flexion angle
Knee angle	Angle between the shank and thigh, flexion-extension angle
Hip angle	Angle between the thigh and pelvis, flexion-extension angle
**Segment angles (°)**	**Kick-leg segment angles measured at BC**
Shank angle	Shank angle about the global y-axis
Thigh angle	Thigh angle about the global y-axis
Pelvis angle	Pelvis angle about the global y-axis
Trunk angle	Trunk angle about the global y-axis
**Angles (°)**	**Direction (vector path, °)**
Foot-path	Angle defined by the linear velocity vector of the kick foot and the line between the foot and the global goal centre in the X-Y plane
*Follow through phase*	
Leg position	Angle between hip and ankle joint about the local z-axis to indicate the ‘straightness of the follow through’
Ankle angle	Angle between the foot and shank, plantar-dorsi flexion angle

### Statistical analysis

Data were divided into accurate (hit, n = 154) and inaccurate (miss, n = 116) kicks. Differences between accurate and inaccurate goal-kicks were assessed using the general linear mixed-model procedure (Proc Mixed) in the Statistical Analysis System studio (version 9.4, SAS 186 Institute, Cary NC). The fixed effects in the model were kick number (five levels, to estimate habituation effects), position (left, right and centre), and accuracy (Hit and Miss). The random effects, estimated as independent variances and allowing for negative variance, were subject identity (between-subject differences), kick position within subjects (within-subject differences between kick position), kick number within kick position (within-subjects changes between kicks) and residuals for each position. Low intra-class correlation coefficients (ICC: <0.12) were reported for the residuals for positions. As a result, kicks were grouped across the three positions to increase kick number and statistical power of the sample, supporting previous research [19]. All data showed no obvious non-uniformity of error. Mean differences ± 90% confidence limits and effect sizes (Cohen’s *d*; small >0.2, medium >0.5, large >0.8) [22] were derived to assess the magnitude of difference between accurate and inaccurate kicks for each technical parameter.

To examine the relationship between the horizontal distance from the goal centre and each parameter, linear (first—order), quadratic (second—order) and cubic (third—order) polynomial curves were calculated. The choice of which curve fit best described the relationship (linear, second- or third-order polynomial) was based on R^2^ values, *p-*value, visual inspection of residual plots, standard error of the estimates (SEE) and the statistical test presented by Hayes [23]. The statistical test from Hayes [23] was important to provide objectivity. Thresholds for interpreting *R*^2^ relationship were, <0.2, no relationship; >0.3 low; 0.50–0.74, moderate; > 0.75, strong [24, 25].

## Results

**Fig 3:**
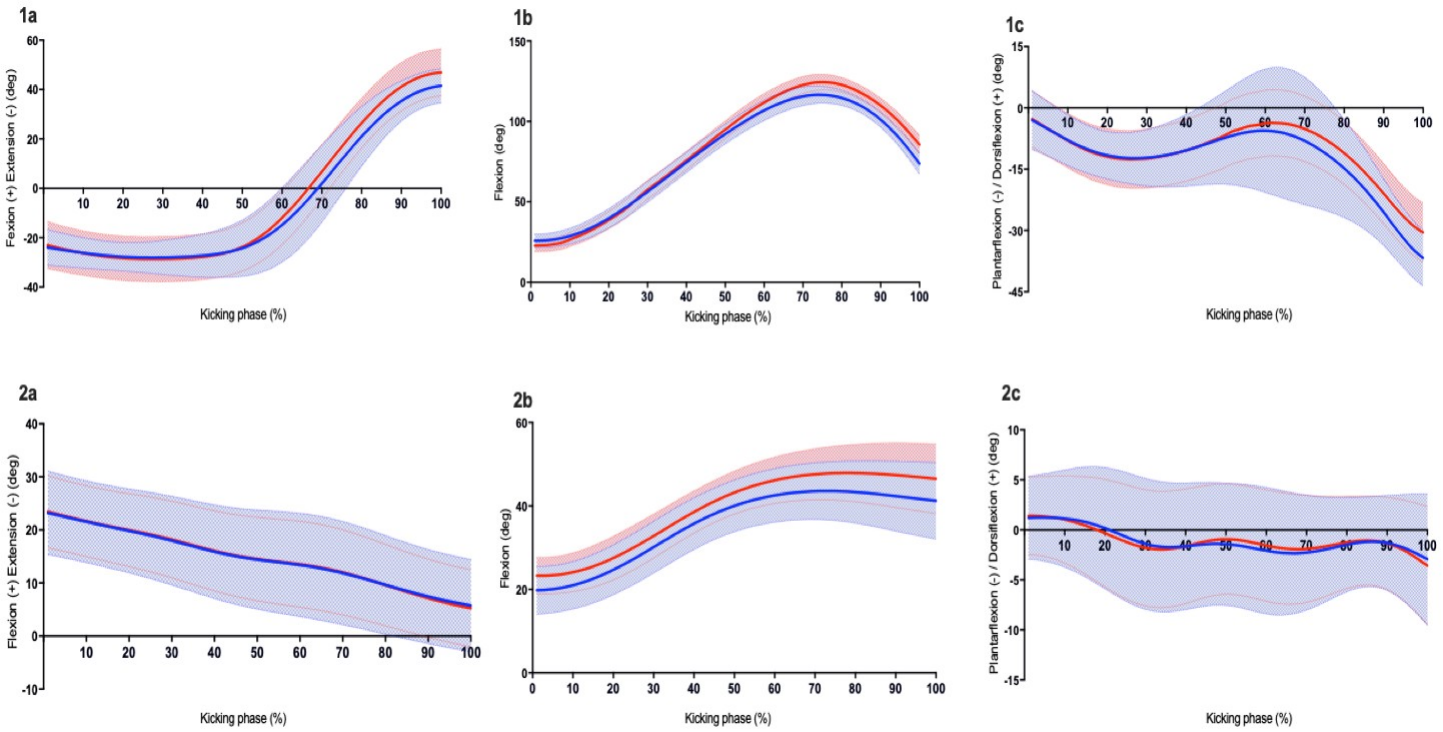
Group mean ± SD for sagittal hip (a), knee (b) and ankle (c) joint angles curves of the kick-leg (1) and support-leg (2) for accurate (blue line) and inaccurate (red line) goal-kicks during the kicking phase (kick leg toe-off: 0%, to BC: 100%).

During the approach phase, a straighter approach line (3 vs 12°, large effect), with small differences in the length of the last step (1.42 vs 1.5 m) and COM velocity at kick-foot toe-off (3.3 vs 3.6 m) were evident during accurate goal-kicks compared to inaccurate goal-kicks (Table 2). During the kicking phase, greater ankle plantar flexion (39 vs 30°, large effect), lower knee (64 vs 69°, large effect) and hip (64 vs 69°, large effect) flexion, with lower linear (foot speed, COM) and angular (ankle, knee and shank) velocities in the kick-leg at BC, were found in accurate kicks. Additionally, accurate goal-kicks demonstrated lower ankle, knee and hip joint range of motion (ROM) in the in the kick-leg throughout the kicking phase (see Table 2 and Fig 3). Support-leg characteristics differed between accurate and inaccurate goal-kicks; accurate kicks demonstrated lower maximum knee flexion at (43 vs 49°, medium effect), which was maintained through to BC (38 vs 43°, large effect) (see Fig 3). At the end of follow through, players finished with a straighter-leg line in the direction of goals (2 vs 12°, large effect), with greater ankle plantarflexion (26 vs 20°, medium effect) during accurate goal-kicks compared to inaccurate goal-kicks.

**Table 2:** Kinematic means ± standard deviations (SD) for accurate (hit) and inaccurate (miss) goal-kicks, mean differences between goal-kicks (Hit-Miss), with 90% confidence limits (CL), effect size (*d*), with 90% CL, and the magnitude of the effect for each parameter. All parameters relate to the kick-leg unless stated.

	Accurate	Inaccurate	Mean Difference,	Effect size (*d*),	Magnitude of effect
Parameter	Mean ± SD	Mean ± SD	90% CL	90% CL	
**Approach phase**					
Last step distance (m)	1.42 ± 0.26	1.52 ± 0.30	-0.10, 0.12	-0.36, 0.52	Small
Average COM velocity (m.s^-1^)	1.9 ± 0.6	1.9 ± 0.4	-0.0, 0.2	-0.03, 0.23	
Max COM velocity (m.s^-1^)	4.1 ± 1.5	4.2 ± 1.3	-0.1, 0.6	-0.07, 0.36	
COM velocity at kick-foot toe-off (m.s^-1^)	3.3 ± 1.4	3.6 ± 1.7	-0.3, 0.7	-0.19, 0.22	Small
Approach angle (°)	3 ± 4	12 ± 3	-9, 2	-1.69, 0.21	Large
**Kicking phase**					
*At Ball Contact*					
Ankle plantar-flexion (°)	39 ± 10	29 ± 6	9, 4	1.20, 0.18	Large
Knee flexion (°)	64 ± 6	69 ± 6	-5, 2	-0.91, 0.21	Medium
Hip flexion (°)	35 ± 10	40 ± 8	-5, 4	-0.63, 0.36	Medium
Pelvic posterior tilt (°)	49 ± 14	48 ± 15	1, 7	0.06, 0.70	-
Trunk posterior tilt (°)	2 ± 9	3 ± 11	-1, 5	-0.07, 0.86	-
Shank angle (°)	-1 ± 10	-5 ± 9	4, 2	0.52, 0.27	Medium
Thigh angle (°)	57 ± 11	58 ± 10	-1, 2	-0.10, 0.19	-
Foot speed (m.s^-1^)	18.0 ± 1.8	19.4 ± 1.4	-1.4, 0.7	-0.89, 0.45	Medium
COM velocity (m.s^-1^)	2.3 ± 0.4	2.7 ± 0.3	-0.4, 0.2	-1.20, 0.28	Large
Knee angular velocity (°/s)	1433 ± 218	1542 ± 202	-109, 93	-0.64, 0.32	Medium
Hip angular velocity (°/s)	56 ± 97	78 ± 100	-18, 45	-0.22, 0.42	Small
Shank angular velocity (°/s)	1647 ± 123	1723 ± 132	-76, 38	-0.63, 0.29	Medium
Thigh angular velocity (°/s)	136 ± 106	154 ± 113	-38, 37	-0.22, 0.19	Small
Ankle angular velocity (°/s)	345 ± 131	433 ± 120	-88, 48	-0.84, 0.45	Medium
Support-leg ankle angle (-plantar/ +dorsi flexion) (°)	-1 ± 7	1 ± 5	-2, 1	0.10, 0.90	-
Support-leg knee flexion (°)	38 ± 5	48 ± 7	-10, 2	-1.21, 0.30	Large
Support-leg hip flexion (°)	15 ± 12	15 ± 11	0, 5	0.00, 0.76	-
	Accurate	Inaccurate	Mean Difference,	Stand. Effect,	Magnitude of effect
*Parameter*	Mean ± SD	Mean ± SD	90% CL	90% CL	
**Kicking phase**					
*Support Heel Strike*					
Support-leg ankle dorsiflexion (°)	20 ± 25	19 ± 11	1, 8	0.05, 0.10	-
Support-leg knee flexion (°)	23 ± 6	25 ± 4	-2, 3	-0.40, 0.55	Small
Support-leg hip flexion (°)	30 ± 9	32 ± 11	-5, 4	-0.19, 0.21	-
*Maxima & Minima*					
Maximum knee flexion (°)	116 ± 13	120 ± 14	-3, 6	-0.29, 0.36	Small
Maximum support-leg knee flexion (°)	43 ± 7	49 ± 7	-5, 3	-0.87, 0.28	Medium
Maximum hip extension (°)	29 ± 6	31 ± 6	-2, 3	-0.36, 0.25	Small
*Range of Motion*					
Ankle ROM (°)	32 ± 4	38 ± 8	-6, 2	-1.23, 0.12	Large
Knee ROM (°)	50 ± 7	54 ± 9	-4, 4	-0.61, 0.34	Medium
Hip ROM (°)	34 ± 9	40 ± 9	-6, 4	-0.69, 0.42	Medium
Pelvis ROM (°)	46 ± 14	48 ± 19	-2, 7	-0.11, 0.23	-
*Direction (vector path*, *°)*					
Foot path angle at BC	0 ± 2	3 ± 4	-3, 1	-0.92, 0.19	Medium
**Follow through phase**					
Leg position at end of follow through(°)	2 ± 7	12 ± 9	-10, 3	-1.24, 0.20	Large
Ankle plantarflexion at end of follow through (°)	10 ± 10	9 ± 15	-6, 6	0.63, 0.23	Medium

Effect size: *d* < 0.2 = none, d < 0.5 = small, d < 0.8 = medium and d > 0.8 = large (Cohen, 1988).

After choosing the most appropriate fit for each relationship, there were six strong (five linear and one quadratic), eight moderate (seven quadratic and one cubic) and twelve low (six quadratic, one linear and five cubic) relationships identified (Table 3). For the remaining parameters, no relationships identified with accuracy.

**Table 3:** The relationship between kinematic parameters and accuracy. Linear, quadratic and cubic curve estimations for each parameter (*r*^*2*^ values (SEE)), with the chosen relationship and magnitude of relationship identified. All parameters relate to the kick-leg unless stated.

Parameter	Relationship			Chosen relationship	Equation	Magnitude of relationship
	1^st^ order	2^nd^ order	3^rd^ order			
**Approach phase**						
Last step distance (m)	0.21 (0.1)	0.24 (0.1)	0.24 (0.1)	quadratic	*y = -0*.*0023x*^*2*^ *+ 0*.*0179x + 1*.*4686*	Low
Average COM velocity (m.s^-1^)	0.02 (0.5)	0.02 (0.4)	0.03 (0.4)	cubic	*y = -0*.*0022x*^*3*^ *+ 0*.*046x*^*2*^*–0*.*1656x + 3*.*4175*	-
Max COM velocity (m.s^-1^)	0.29 (0.4)	0.31 (0.4)	0.35 (0.4)	linear	*y = 0*.*0157x + 4*.*2724*	Low
COM velocity at KFTO (m.s^-1^)	0.27 (0.3)	0.31 (0.3)	0.39 (0.3)	cubic	*y = -0*.*0114x*^*3*^ *+ 0*.*1264x*^*2*^ *+ 0*.*716x + 3*.*4398*	Low
Approach angle (°)	0.83 (1.1)	0.79 (1.2)	0.63 (1.2)	linear	*y = 1*.*4178x + 0*.*43*	Strong*
**Kicking phase**						
*At Ball Contact*						
Ankle plantar-flexion (°)	0.62 (2.4)	0.68 (1.9)	0.65 (2.2)	quadratic	*y = 0*.*1515x*^*2*^ *+ 1*.*3959x − 41*.*911*	Strong*
Knee flexion (°)	0.56 (2.7)	0.65 (2.3)	0.68 (2.1)	cubic	*y = -2*.*17x*^*3*^ *+ 0*.*1663x*^*2*^ *+ 1*.*6096x + 58*.*358*	Moderate*
Hip flexion (°)	-0.02 (5.4)	0.03 (4.8)	0.03 (4.6)	cubic	*y = 0*.*0016x*^*3*^*–0*.*0069x*^*2*^ *+ 0*.*0638x + 2*.*6678*	-
Pelvic posterior tilt (°)	-0.01 (4.4)	0.03 (4.3)	0.16 (4.3)	cubic	*y = 0*.*0386x*^*3*^*–0*.*766x*^*2*^ *+ 3*.*5616x + 55*.*435*	-
Trunk posterior tilt (°)	-0.18 (1.0)	0.18 (1.2)	0.15 (1.2)	linear	*y = 0*.*1312x + 1*.*5981*	-
Shank angle (°)	0.57 (4.8)	0.59 (4.8)	0.59 (4.8)	quadratic	*y = -0*.*0464x*^*2*^*–0*.*0126x − 1*.*012*	Moderate*
Thigh angle (°)	0.25 (3.7)	0.25 (3.6)	0.22 (3.6)	quadratic	*y = -1*.*362x*^*2*^ *+ 1*.*4825x + 54*.*946*	Low
Foot speed (m.s^-1^)	0.83 (0.6)	0.80 (0.7)	0.81 (0.8)	linear	*y = 0*.*8282x + 17*.*186*	Strong*
COM velocity (m.s^-1^)	0.33 (0.2)	0.35 (0.1)	0.38 (0.1)	cubic	*y = -0*.*002x*^*3*^ *+ 0*.*0307x*^*2*^*–0*.*0687x + 2*.*3012*	Low
Knee angular velocity (°/s)	0.20 (67)	0.27 (67)	0.26 (68)	quadratic	*y = 0*.*3507x*^*2*^*–16*.*266x + 1528*	Low
Hip angular velocity (°/s)	0.26 (28)	0.27 (27)	0.24 (27)	quadratic	*y = -0*.*236x*^*2*^ *+ 9*.*2787x + 122*.*58*	Low
Shank angular velocity (°/s)	0.52 (43)	0.53 (43)	0.53 (43)	quadratic	*y = -0*.*3967x*^*2*^ *+ 6*.*6706x + 1634*.*9*	Moderate
Thigh angular velocity (°/s)	0.30 (32)	0.37 (32)	0.36 (32)	quadratic	*y = -0*.*7366x*^*2*^ *+ 9*.*0505x + 93*.*359*	Low
Ankle angular velocity (°/s)	0.63 (38)	0.67 (37)	0.67 (37)	quadratic	*y = -0*.*0898x*^*2*^ *+ 2*.*4336x − 0*.*6136*	Moderate*
SL ankle angle (°)	0.09 (1.0)	0.09 (1.0)	0.08 (1.0)	cubic	*y = 0*.*0014x*^*3*^*–0*.*0424x*^*2*^ *+ 0*.*293x − 0*.*0433*	-
SL knee flexion (°)	0.64 (1.5)	0.72 (1.4)	0.72 (1.4)	quadratic	*y = -0*.*5339x*^*2*^ *+ 6*.*682x + 28*.*226*	Moderate*
SL hip angle (°)	0.28 (1.1)	0.37 (1.1)	0.37 (1.1)	cubic	*y = 0*.*0103x*^*3*^ *+ 0*.*265x*^*2*^*–0*.*4593x − 36*.*939*	Low
*At Support Heel Strike*						
SL ankle dorsiflexion (°)	-0.03 (1.9)	0.05 (1.9)	0.05 (1.9)	cubic	*y = -0*.*006x*^*3*^*–0*.*005x*^*2*^ *+ 1*.*3992x + 12*.*896*	-
SL knee flexion (°)	0.08 (1.5)	0.09 (1.5)	0.09 (1.5)	cubic	*y = -0*.*004x*^*3*^ *+ 0*.*044x*^*2*^ *+ 0*.*2686x + 18*.*927*	-
SL hip flexion (°)	0.02 (0.8)	0.05 (0.8)	0.06 (0.8)	cubic	*y = -0*.*0166x*^*3*^ *+ 0*.*2095x*^*2*^*–0*.*015x + 27*.*133*	-
*Maxima & Minima*						
Max knee flexion (°)	0.21 (1.6)	0.27 (1.6)	0.29 (1.6)	cubic	*y = -0*.*0088x*^*3*^ *+ 0*.*1092x*^*2*^ *+ 0*.*1841x + 115*.*21*	Low
Max SL knee flexion (°)	0.34 (2.2)	0.60 (2.1)	0.60 (2.1)	quadratic	*y = -0*.*2753x*^*2*^ *+ 3*.*7763x + 29*.*233*	Moderate*
Max hip extension (°)	0.19 (2.5)	0.22 (2.4)	0.33 (2.4)	cubic	*y = 0*.*0157x*^*3*^*–0*.*3191x*^*2*^ *+ 1*.*2419x − 30*.*298*	Low
*Range of Motion*						
Ankle ROM (°)	0.43 (1.3)	0.56 (1.3)	0.56 (1.3)	quadratic	*y = -0*.*1594x*^*2*^ *+ 2*.*5691x + 28*.*164*	Moderate*
Knee ROM (°)	0.14 (2.3)	0.24 (2.3)	0.25 (2.3)	quadratic	*y = 0*.*0079x*^*3*^*–0*.*2184x*^*2*^ *+ 1*.*8215x + 89*.*04*	Low
Hip ROM (°)	0.52 (2.4)	0.75 (2.3)	0.69 (2.4)	quadratic	*y = -0*.*3165x*^*2*^ *+ 5*.*3642x + 26*.*296*	Strong*
Pelvis ROM (°)	0.02 (3.0)	0.03 (3.1)	0.03(3.2)	cubic	*y = -0*.*016x*^*3*^ *+ 0*.*2964x*^*2*^*–0*.*8621x + 36*.*517*	-
*Direction (vector path*, *°)*						
Foot path angle at BC	0.92 (0.2)	0.89 (0.5)	0.84 (0.5)	linear	y = 0.5899x + 0.0155	Strong*
**At the end of the Follow through phase**						
Leg position (°)	0.73 (1.7)	0.73 (1.8)	0.73 (1.8)	linear	*y = 56*.*67x + 2*.*491*	Strong*
Ankle plantarflexion (°)	0.75 (2.4)	0.73 (2.5)	0.73 (2.5)	linear	*y = 1*.*6024x − 1*.*977*	Strong*

For linear relationships, a negative sign denotes a negative of relationship.

For linear relationships, a negative sign denotes the direction of the relationship. **SL**: support leg; **SEE**: standard error of the estimate; **COM**: centre of mass.

* indicate p<0.05.

## Discussion

The purpose of this research was to examine goal-kicking technique in AF and determine technical factors associated with accuracy. Findings indicated that many factors interact with accurate goal-kicking in AF, ranging from the player’s approach line, kick-leg swing motion, support-leg mechanics, through to their final position at the end of the follow through.

**Fig 4:**
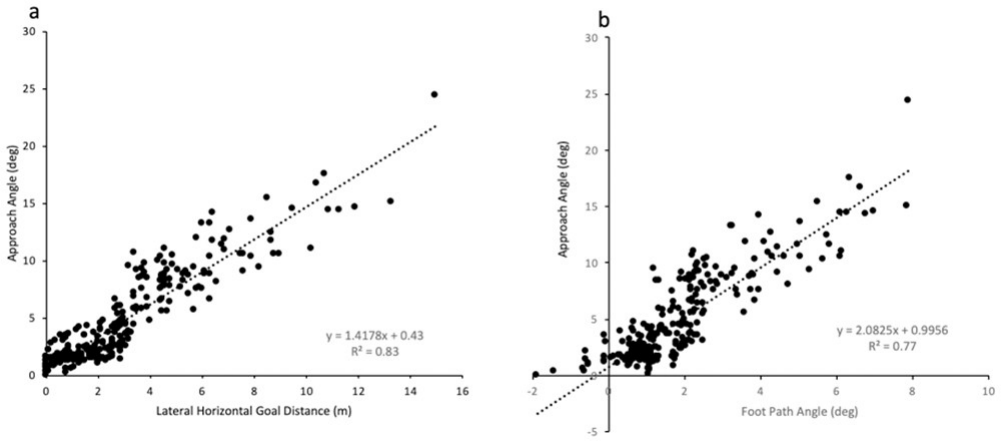
Relationship between (a) approach angle and accuracy (values to the right of the dashed indicate missed kicks) and (b) approach angle and foot-path angle.

The angle of a player’s approach line is an important factor for accurate goal-kicking. A straighter approach line was evident in accurate goal-kicks compared to inaccurate goal-kicks, with a strong linear relationship reported between approach angle and accuracy (Fig 4). These findings are in agreement with previous scientific findings [3, 5] and coaching recommendations [4]. Adopting a straighter line of approach is suggested to increase the planarity of the goal-kick action, through limiting the rotation of the kick-leg around the vertical axis through the body [3, 5, 26]. This in turn, enables players to apply a straighter striking force on the ball relative to the goal centre [3], which according to the oblique impact theory directly influences the ball’s flight characteristics [25, 27]. Post-hoc analysis supported this concept by indicating a strong relationship between foot-path angle at BC (smaller frontal foot-path angle reflects a more direct line of contact with the ball) and approach angle (*r*^*2*^ = 0.77) (Fig 4). Theoretically, a more direct (less angled) striking force applied close to the ball’s centre of mass would propel the ball straight towards the target, with minimal medio-lateral spin (side spin) [8, 12, 26, 28, 29]. As a result, this would reduce the lateral deviation of the ball’s flight trajectory away from the centre of the target [26]. Interestingly, no kicks were missed when the approach angle was less than 4.7° (Fig 4), emphasising the benefit of players adopting a straighter line of approach.

**Fig 5:**
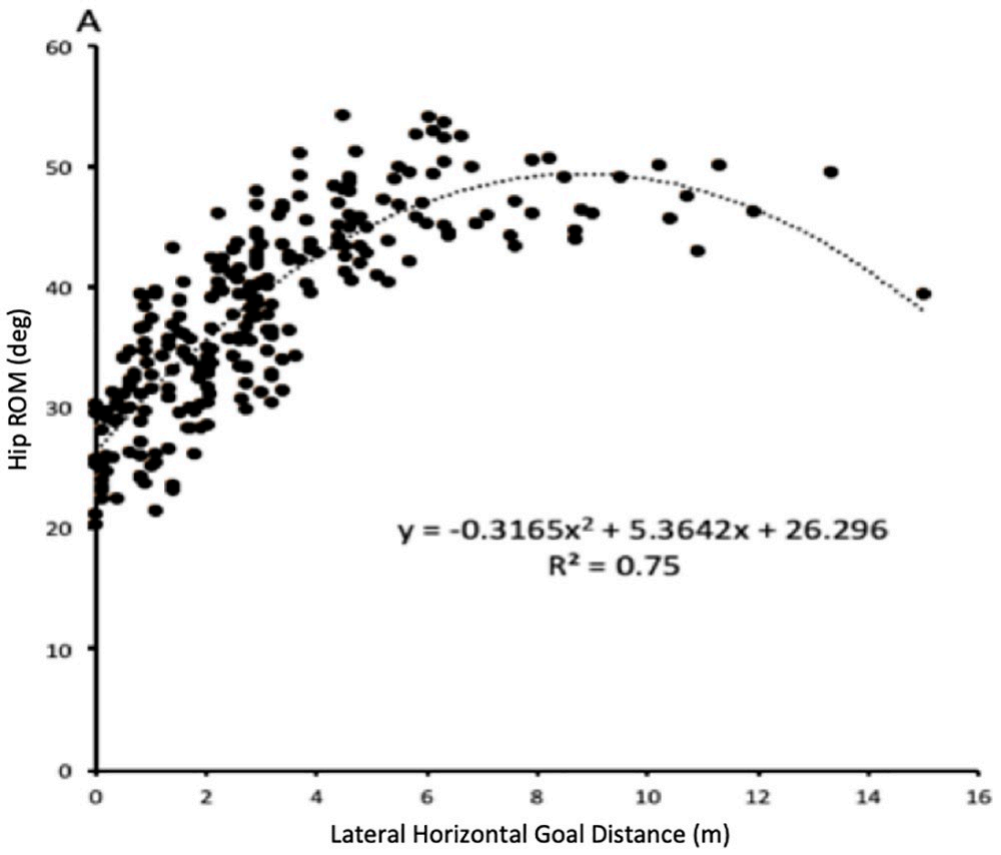
The relationship between accuracy and hip ROM; (a) strong quadratic relationship between hip ROM and accuracy.

Accurate goal-kicking requires control and regulation of the kick-leg motion during the kicking phase. Accurate goal-kicks were associated with moderately less hip and knee ROM, with slower knee and shank angular velocities throughout the kicking phase, supporting previous findings [16, 17, 29]. Two possibilities exist for this reduced ROM/speed strategy. Firstly, reduced ROM at the kick-leg joints might support a strategy of reducing movement speed, representative of the speed-accuracy trade-off, or Fitts’s law [30]. This theory identifies an inverse relationship between the speed at which a skill can be performed and the accuracy that can be achieved [30]. This trade-off may have prompted the changes in hip and knee ROM during accurate goal-kicks, as reductions in the speed of a movement is fundamentally linked to lower ROM [31, 32]. Secondly, players might be constraining the hip/knee ROM to increase control and regulation of the motion of the kicking limb, in an attempt to better position and orientate kicking limb for ball impact [16, 33]. This would be representative of the freezing of the redundant degrees of freedom (DOF) in a task-specific functional way [34–39]. Where the nervous system may arrive at the desired movement solution by reducing the full range of a specific task-relevant biomechanical variable (i.e., knee and hip ROM) [38]. However, a strong quadratic relationship (*r*^*2*^ = 0.75) was identified between hip ROM and accuracy (Fig 5), indicating that an optimal point of hip movement for goal-kicking accuracy exists. While reduced hip ROM might support a strategy to improve accuracy, it appears there is a point at which insufficient ROM may constrain the movement of the kick-leg.

While initial joint configuration of the hip, knee and ankle at the start of the kicking phase did not differ between accurate and inaccurate goal-kicks, substantial alterations in joint motions occurred in the final phase (60–100%) of the movement (Fig 3). In accurate kicks, players exhibited a more extended posture (greater ankle plantarflexion, knee and hip extension in the kick-leg, with a more extended support-leg) at the BC, supporting previous findings [33, 40]. It is logical to suggest that players may make active adjustments to kick-leg mechanics during the final part of the kicking phase, to compensate for changes in ball drop position (i.e. accounting for ball drop error). This is an important feature of the concept of motor abundance and functional synergy [38]; where if the contribution of one component (i.e., ball drop characteristics) at a particular time has a perturbing effect on an important performance variable (i.e. impact characteristics), other components are likely to modify their contributions to stabilise the performance outcome. Future research is warranted to examine the technique of controlling and dropping the ball, along with the interceptive task of striking the foot and various non-linear combinations of parameters, as it may provide an additional insight of important factors which influence goal-kicking in AF.

**Fig 6:**
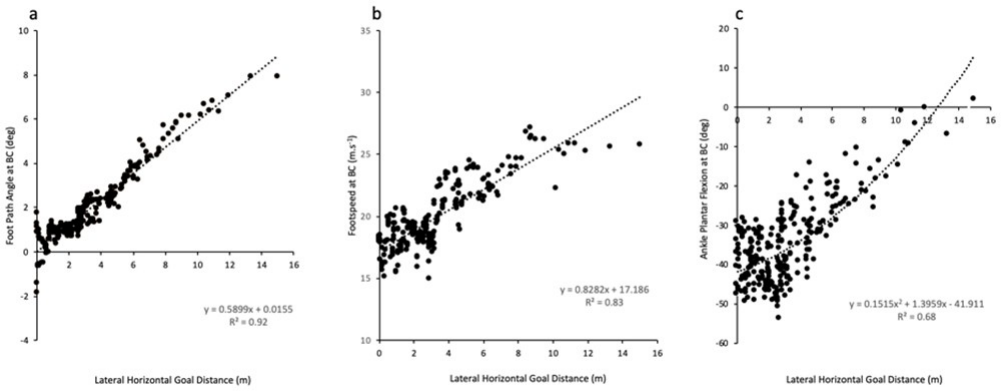
The relationship between accuracy and (a) footpath angle at BC, (b) foot speed at BC, (c) ankle plantarflexion at BC.

Ankle and foot motion play a vital role in the success of a goal-kick. Players had lower ankle ROM, foot speeds and ankle angular velocity, with higher ankle plantar flexion and a straighter foot-path at BC during accurate kicks. Additionally, strong linear relationships were reported in footspeed and foot-path angle at BC, with a moderate quadratic relationship identified for ankle plantarflexion at BC (Fig 6). Several possibilities exist for the ankle and foot strategy. Firstly, decreased ankle ROM and angular velocity, along with slower foot speeds may be utilised to increase stabilisation and control of the foot in preparation for BC [16]. Controlling foot motion so it is in an optimal position for impact, would enable players to impart the desired flight characteristics on the ball to achieve a successful outcome [41, 42]. This adjustment would represent a task dependent freezing of the redundant DOF, in order to attempt to stabilise the performance outcome [34, 39, 38] but may be also representative of the speed-accuracy trade-off [30]. Secondly, increasing ankle/ foot segment rigidity has been associated with increased impact efficiency and accuracy [42–46], which can be achieved through increasing ankle plantarflexion prior to and through ball impact [8, 42, 43, 45–48]. Increased ankle joint plantarflexion has also been suggested to enable players to reduce the uneven pressures across the anterior aspect of the foot (caused by bony prominences) and apply a more homogenous force to the ball to achieve a straighter ball flight trajectory [11, 45]. Lastly, players may be actively controlling the motion of the kick-foot to ensure a straighter line of force is applied to the ball, through having a more direct line of contact (a smaller foot-angle at BC). This provides biomechanical support the coaching cue “strike through the ball in the direction of the target” [4]. It may be that a combination of each of these adjustments in the distal segment are required to improve goal-kicking accuracy in AF.

**Fig 7:**
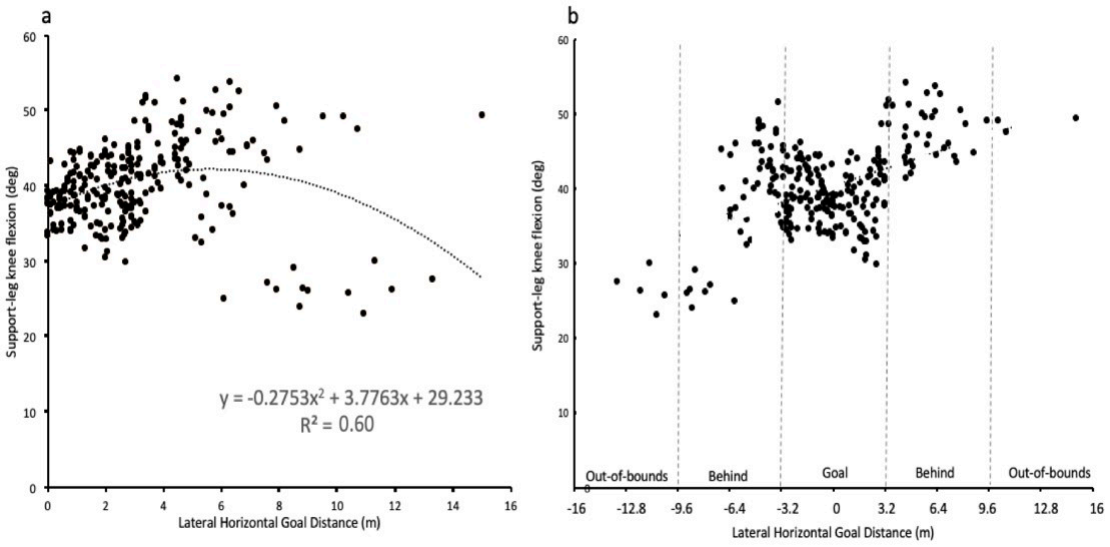
The relationship between accuracy and support-leg knee flexion; (a) a moderate quadratic relationship between support-leg knee flexion and accuracy, and (b) alterations in support-leg knee flexion in relation to left and right side of goals.

Support-leg knee motion is important for kicking accuracy in AF. Players demonstrated a more extended (less flexion) knee at SHS that remained more extended during the stance phase until BC compared during accurate goal-kicks (Fig 3), supporting previous findings in distance kicking [7]. Ball [7] suggested this could be indicating a stronger and more stable stance-leg during the kick-action. Increasing stability is suggested to be a fundamental prerequisite in the organisation of a skilled movement, in order to improve accuracy [49–51]. Greater stabilisation of the support-leg would provide the kicker with a stronger base of support to facilitate better control and regulation of the kick-leg motion during the kicking phase [7, 33, 51, 52]. A moderate quadratic relationship was identified between support-leg knee flexion and accuracy (Fig 7). Further examination identified there was a trend with support-leg knee flexion and missing to the right or left of goals (Fig 7). Players had a tendency to display less knee flexion when they missed to the left of goals, while greater knee flexion was evident when players missed to the right of goals. One possible explanation for this may be related to alterations in swing plane characteristics [12, 26]. Having a more extended position (less knee flexion) would allow more rotation of the kick-leg around the vertical axis, resulting in a more curved movement path of the kick-leg. This would potentially result in a more lateral impact location, which in turn would cause a more medio-lateral spin on the ball [8], causing the ball’s flight path to deviate left of the target centre [3, 26, 32]. Findings by Alcock et al. [26] support this possibility as a more curved ball flight path trajectory when the kicking-leg swing plane was steeper. Based on these findings, coaches working with kickers who have a tendency to miss to the left or right of the target, could aim at altering support-leg mechanics (either promoting an increase in knee flexion or decrease depending on the player’s performance) as a potential avenue for improvement.

However, the findings in this study are in contrast to the findings that a more flexed support-leg is better for kicking accuracy in general play kicks in AF [9, 10]. A possible reason for the conflicting findings may be directly related to the shorter distances used between the accuracy tasks (15 m [10]; 20 m [9]) compared to the distance (30 m) used in this study. Researchers have reported that when kicking distance increases, players are required to increase foot speed and ball speed accordingly, in order to meet the distance demand [3, 6, 11, 53]. Lifting the whole-body upward through the motion of the support-leg (through knee extension) has been identified as an effective action to help generate faster foot speed’s through achieving a more extended kick-leg (and hence a longer lever arm) during the swing phase [7, 51, 54]. This explanation is partly supported by the higher foot speed’s (18.0 m.s^-1^) reported in this study compared those reported from Blair et al. [9] (13.2 m.s^-1^). Another possible explanation may be that when kicking over shorter distances players might have purposely attempted to increase the relative target area by adopting a flatter ball flight trajectory to improve accuracy [11]. A lower ball flight trajectory would be achievable though adopting a more flexed kicking position [11], which could be partly achieved through increased support-leg flexion. Conversely, when kicking at further distances from goals, achieving a higher ball flight trajectory (lofted kick) may be more beneficial to achieve the distance, as well as ensuring accuracy. This may not be surprising given that alterations in the task constraints (such as, the distance of the goal-kick) have been found to trigger substantial differences in the way posture is organised to facilitate movement when achieving the same performance outcome [21, 55]. These findings may be indicative that variations in the task constraints leads to significant changes in the movement pattern required to complete the task. It is possible that this represents a continuum of technique strategy, where at one end (short kicks for accuracy) a more flexed support leg is beneficial while at the other end (maximising distance) a more extended support leg is beneficial. Examination of accurate goal-kicking technique over a range of distances would provide important information on how players adapt to different task constraints.

The motion of the kicker through the follow-through phase is suggested to indicate the motion path and the kinematics of the kick-leg prior to impact [3, 56] and is a common point of focus for coaching. Supporting previous research [3, 5], players finished with their leg in-line towards the target, with greater ankle plantarflexion at the end of follow through during accurate kicks, whilst player’s had a tendency to swing their leg across the mid-line of the body, with less ankle plantarflexion in inaccurate kicks. Finishing with the toe pointing towards the goals is suggested to reflect a more planar swing motion of the kick-leg during the kicking phase [3]. It is logical to assume that if a player increases the planarity of the kick-leg motion during the kicking phase, the kick-leg would follow in a similar motion path during the follow through. In contrast, swinging the kick-leg across the mid-line of the body would indicate that kick-leg followed a curved path (greater rotation of the kick-leg around the vertical axis) during the kicking phase. Additionally, higher ankle plantarflexion during the follow-through is suggested to provide an indication that players maintained a more rigid ankle through the kicking phase [4], to improve accuracy [8, 39, 43, 45, 47]. These findings provide scientific evidence to support the appropriateness and potential influence of the currently used coaching cue “finish with your toe pointing towards goals” [4].

A group-based analysis approach was utilised to characterise goal-kicking technique to help establish an evidence base to better define the key technical factors that are associated with goal-kicking performance in AF. Given the advantages of a group-based approach (i.e. provides a larger sample to control for inter-subject variability to provide adequate statistical power), the results can be generalised to the larger population [57] to develop a general understanding of what ‘good’ goal-kicking technique resembles. This information can then be used to objectively guide development programmes designed at improving goal-kicking performance across a range of levels. Given that research has also identified the possibility of the existence of individual-specific finding in AF kicking [6, 7, 9, 19, 58], further work is required to investigate the presence of individual-specific strategies in goal-kicking through the use of an individual-based analysis approach. Furthermore, goal-kicking technique was examined under a non-fatigued state in a training environment, which enabled an ecologically valid examination of technique under ‘ideal’ conditions. However, players also need to successfully adapt to numerous other fluctuating constraints during competition such as, task constraints (fatigue), environmental constraints (wind, rain, crowd noise), personal constraints (anxiety, decision-making skills) and contextual factors (finals, score margin and time remaining) [1]. Theoretical frameworks such as, the dynamical systems theory [59, 60] may offer a useful framework for future investigations to examine how the goal-kicking skill is affected by these constraints.

Based on the current findings, a number of practical implications exist for the coaching of the goal-kicking skill:

- Instructing players to adopt a straight line of approach and “finish with their toe pointing towards goals” or “strike through the ball in the direction of the target” may help increase the accuracy and planarity of goal-kicking movement.
- ‘Kick with a firm foot’ may be an effective instruction to encourage players to plantarflex the ankle to improve ankle more rigidity, which in turn assists with impact efficiency [4]. Players with low ankle rigidity should include strength training in order to improve this aspect of kicking [7].
- Conditioning the support-leg to maintain an extended position may assist kickers attain a stronger base of support to facilitate a more controlled kick-leg motion. Use of task-specific movements, such as single-legged landing task and lateral lunges with a knee drive may be effective [7].

## Conclusion

During the approach phase, players demonstrated a substantially straighter approach line during accurate goal-kicks compared to inaccurate goal-kicks. During the kicking phase, accurate goal-kicks were associated with substantially lower kick-leg ankle, knee and hip ROM, a more direct foot path, substantially greater ankle plantar flexion and lower knee flexion, with lower joint (knee) and segment (shank) velocities in the kick-leg at BC compared to inaccurate kicks. Support-leg characteristics differed between accurate and inaccurate kicks; accurate kicks demonstrated lower hip and knee flexion. At the end of follow through, players finished with a straighter-leg line with a greater ankle plantarflexion during accurate kicks compared to inaccurate goal-kicks. In addition, a number of substantial linear and quadratic relationships were reported between technical parameters and accuracy. Many factors were found to interact with accurate goal-kicking in AF, ranging from a players approach line path, their support-leg mechanics, kick-leg swing motion, through to their final position at the end of follow through.
